# Supplementary material for: First Report, Characterization and Pathogenicity of Vibrio chagasii Isolated from Diseased Reared Larvae of Chilean Scallop, Argopecten purpuratus (Lamarck, 1819)
Source: Pathogens. 2023 Jan 24;12(2):183. doi: 10.3390/pathogens12020183 (PMC9964487; doi:10.3390/pathogens12020183)
Supplement: Supplementary file 1 [file pathogens-12-00183-s001.zip › pathogens-2107539-supplementary.pdf]

Table S1. Enzymatic properties of the *Vibrio chagasii* VPAP36 and VPAP40 strains by using the API 20E system (BioMerieux).

Activity	Strain	
	**VPAP36**	**VPAP40**
β-galactosidase	−	–
Arginine dihydrolase	+	+
Lysine decarboxylase	−	−
Ornithine decarboxylase	−	−
Citrate utilization	+	+
H_2S production	−	−
Urease	−	−
Tryptophane deaminase	+	−
Indole production	+	+
Acetoin production	−	−
Gelatinase	+	+
Fermentation of:		
glucose	+	+
mannitol	+	+
inositol	−	−
sorbitol	−	−
rhamnose	−	−
saccharose	+	+
melibiose	−	−
amygdalin	+	+
arabinose	−	−

Table S2. Phenotypic characterization of the *Vibrio chagasii* VPAP36 and VPAP40 strains by using the BIOLOG system.

Carbon source	Strain		Carbon source	Strain	
	VPAP36	VPAP40		VPAP36	VPAP40
α-cyclodextrin	−	−	Itaconic acid	−	−
Dextrin	+	+	α-keto butyric acid	−	−
Glycogen	+	+	α-keto glutaric acid	+	−
Tween 40	−	+	α-keto valeric acid	−	−
Tween 80	−	+	D,L-lactic acid	+	+
N-acetyl-D-galactosamine	−	−	Malonic acid	−	−
N-acetyl-D-glucosamine	+	$+^{w}$	Propionic acid	−	−
Adonitol	−	−	Quinic acid	$+^{w}$	+
L-arabinose	−	−	D-saccharic acid	−	−
D-arabinol	−	−	Sebacic acid	−	−
D-cellobiose	+	+	Succinic acid	+	+
i-erythritol	−	−	Bromo succinic acid	+	−
D-fructose	+	+	Succinamic acid	−	−
L-fucose	−	−	Glucuronamide	−	−
D-galactose	−	−	L-alaninamide	−	−
Gentiobiose	−	+	D-alanine	−	+
α-D-glucose	+	−	L-alanine	+	+
m-inositol	−	−	L-alanyl-glycine	+	+
α-D-lactose	−	−	L-asparagine	+	+
Lactulose	−	−	L-aspartic acid	+	+
Maltose	+	+	L-glutamic acid	+	+
D-mannitol	+	+	Glycyl L-aspartic acid	+	+
D-mannose	+	−	Glycyl L-glutamic acid	+	+
D-melibiose	−	−	L-histidine	$+^{w}$	$+^{w}$
β-metil-D-glucoside	−	−	Hydroxy L-proline	−	−
D-psicose	−	−	L-leucine	−	−
D-raffinose	−	−	L-ornithine	−	$+^{w}$
L-rhamnose	−	−	L-phenilalanine	−	−
D-sorbitol	−	−	L-proline	$+^{w}$	+
Sucrose	+	+	L-pyroglutamic acid	−	−
D-trehalose	+	+	D-serine	−	−
Turanose	−	$+^{w}$	L-serine	+	+
Xylitol	−	−	L-threonine	+	+
Methyl-pyruvate	−	−	D,L-carnitine	−	$+^{w}$
Mono-methyl-succinate	−	−	γ-amino butyric acid	−	−
Acetic acid	$+^{w}$	−	Urocanic acid	−	−
Cis-aconitic acid	−	−	Inosine	+	+
Citric acid	−	−	Uridine	+	+
Formic acid	−	−	Thymidine	+	+
D-galactonic acid lactona	−	−	Phenyethylamine	−	−
D-galacturonic acid	−	−	Putrescine	−	−
D-gluconic acid	−	−	2-aminoethanol	−	$+^{w}$
D-glucosaminic acid	−	−	2,3-butanediol	−	−
D-glucuronic acid	+	−	Glycerol	+	+
α-hydroxy butyric acid	−	−	D,L α-glycerol phosphate	−	+
β-hydroxy butyric acid	−	−	Glucose 1-phosphate	$+^{w}$	+
γ-hydroxy butyric acid	−	−	Glucose 6-phosphate	+	+
p-hydroxy phenylacetic acid	−	−			

+: Positive reaction; $+^{w}$: Weak reaction; −: Negative reaction
